# Response to commentary on “pembrolizumab in gestational trophoblastic neoplasia: systematic review and meta-analysis with sub-group analysis of potential prognostic factors”

**DOI:** 10.1016/j.clinsp.2025.100713

**Published:** 2025-06-27

**Authors:** Marcio Barcellos, Antonio Braga, Matheus Machado Rech, Solange Artimos de Oliveira, Jose Mauro Madi, Sue Yazaki Sun, Jorge de Rezende-Filho, Kevin M. Elias, Neil S. Horowitz, Ross S. Berkowitz

**Affiliations:** a Rio de Janeiro Trophoblastic Disease Center, Maternidade Escola da Universidade Federal do Rio de Janeiro, Rio de Janeiro, RJ, Brazil; b Postgraduate Program in Medical Sciences, Faculdade de Medicina da Universidade Federal Fluminense, Rio de Janeiro, RJ, Brazil; c Postgraduate Program in Applied Health Sciences, Universidade Vassouras, Vassouras, RJ, Brazil; d Caxias do Sul Trophoblastic Disease Center, Faculdade de Medicina da Universidade de Caxias do Sul, Caxias do Sul, RS, Brazil; e Department of Obstetrics. Escola Paulista de Medicina, Universidade Federal de São Paulo, São Paulo, SP, Brazil; f New England Trophoblastic Disease Center, Division of Gynecologic Oncology, Department of Obstetrics, Gynecology and Reproductive Biology, Brigham and Women's Hospital, Dana-Farber Cancer Institute, Harvard Medical School, Boston, MA, USA

Dear editor,

We appreciate the thoughtful commentary by the authors^1^ regarding our systematic review and meta-analysis on pembrolizumab in Gestational Trophoblastic Neoplasia (GTN).^2^ Their constructive feedback underscores important methodological considerations that warrant further discussion.

## Regarding sensitivity analysis based on study quality

We agree that sensitivity analyses based on study quality can yield valuable insights in meta-analyses. In our case, all studies included in our analysis met acceptable quality standards, as assessed using the Newcastle-Ottawa Scale, with none identified as having a critical risk of bias that would justify exclusion.^3^ Given the consistently moderate-to-high quality of the included studies and the limited number available, we concluded that a stratified sensitivity analysis would offer limited benefit and could potentially introduce misleading interpretations.

Excluding studies based on arbitrary quality thresholds would have significantly reduced an already scarce evidence base, without strong methodological justification. This limitation is common in the synthesis of evidence for emerging treatments, particularly when data remains sparse.^4^ We remain confident that our quality assessment approach was both appropriate and sufficient to support the robustness of our findings within the constraints of the available evidence.

## Regarding publication bias assessment

We appreciate the suggestion to incorporate the Luis Furuya-Kanamori (LFK) index and Doi plots in the evaluation of publication bias. While these tools represent emerging methodologies in meta-analysis, recent evidence has raised concerns about their reliability. A comprehensive simulation study by Schwarzer et al. found that the LFK index can yield highly inconsistent results, with a high rate of false positives influenced by factors such as study size, number, and heterogeneity.^5^ The authors explicitly concluded that “the LFK index test in its current implementation should not be used to assess funnel plot asymmetry in meta-analysis”.

In contrast, our approach employed funnel plots in conjunction with Duval and Tweedie’s trim-and-fill method ‒ a well-established and widely accepted technique in meta-analytic practice. Moreover, as outlined in the Cochrane Handbook, statistical tests for funnel plot asymmetry are not recommended when fewer than 10 studies are included, due to their low power and susceptibility to misleading results.^6^ In light of these methodological considerations and the small number of studies in our meta-analysis, we believe our approach to assessing publication bias was both appropriate and methodologically sound.

## Regarding prediction intervals

We acknowledge the thoughtful recommendation to incorporate prediction intervals alongside confidence intervals. However, in meta-analyses with a small number of studies and considerable heterogeneity, as is the case with pembrolizumab for GTN treatment, prediction intervals can be highly unstable and of limited interpretive value.

As discussed by Riley et al.^7^ and Partlett and Riley.^8^ prediction intervals are often poorly estimated when fewer than 10 studies are available. In our specific context, which includes single-arm data drawn from heterogeneous sources (ranging from case reports to small case series), prediction intervals could theoretically span from near 0 % to 100 % response rates. This would offer little actionable insight and may introduce confusion rather than clarity.

Our use of a 95 % Confidence Interval around the pooled estimate, calculated using a random-effects model, remains the most straightforward and reliable method for conveying statistical uncertainty. It appropriately captures the precision of the combined estimate while accounting for between-study variability, which is particularly relevant in evidence synthesis with limited data^9^.

## Regarding molecular markers in subgroup analysis

We fully agree with the suggestion to explore molecular and immunological markers as prognostic indicators of response. Emerging evidence indicates that the Programmed Death-Ligand-1 (PD-L1) expression may play a critical role in predicting response to pembrolizumab in GTN. For instance, studies have demonstrated that choriocarcinoma and related tumors frequently exhibit strong PD-L1 expression, which is associated with favorable responses to checkpoint inhibitor therapy.^10^

Regrettably, among the studies included in our review, molecular and immunological characteristics were rarely and inconsistently reported. Consequently, our subgroup analyses were necessarily restricted to patient-level variables that were uniformly documented across the data set. Nevertheless, we strongly endorse the perspective that future studies should prioritize the inclusion of such biomarkers. Greater insight into the molecular determinants of response could significantly advance the development of personalized treatment strategies for GTN.

## Conclusion

Following careful consideration of the commentary, we reaffirm that the methodological approaches adopted in our meta-analysis were appropriate given the constraints of the available data. While the proposed additional analyses could offer complementary perspectives, they would not materially alter the results or undermine the validity of our conclusions. Our central finding, that pembrolizumab demonstrates promising efficacy in chemoresistant/relapse GTN, remains robust.

We are grateful for this scholarly dialogue, which raises important methodological considerations in evidence synthesis for emerging therapies. Such exchanges contribute meaningfully to the advancement of knowledge and ultimately serve to improve outcomes for patients facing this challenging condition.

## Declaration of competing interest

The authors declare no conflicts of interest.
